# Pigmentation Chemistry and Radical‐Based Collagen Degradation in Alkaptonuria and Osteoarthritic Cartilage†

**DOI:** 10.1002/anie.202000618

**Published:** 2020-05-14

**Authors:** Wing Ying Chow, Brendan P. Norman, Norman B. Roberts, Lakshminarayan R. Ranganath, Christian Teutloff, Robert Bittl, Melinda J. Duer, James A. Gallagher, Hartmut Oschkinat

**Affiliations:** ^1^ Leibniz-Forschungsinstitut für Molekulare Pharmakologie, im Forschungsverbund Berlin e.V. (FMP) Campus Berlin-Buch, Robert-Rössle-Str. 10 13125 Berlin Germany; ^2^ Department of Musculoskeletal Biology Institute of Ageing & Chronic Disease William Henry Duncan Building University of Liverpool Liverpool L7 8TX UK; ^3^ Departments of Clinical Biochemistry and Metabolic Medicine Royal Liverpool and Broadgreen University Hospitals Trust Liverpool L7 8XP UK; ^4^ Freie Universität Berlin Fachbereich Physik, Berlin Joint EPR Lab Arnimallee 14 14195 Berlin Germany; ^5^ Department of Chemistry University of Cambridge Lensfield Road Cambridge CB2 1EW UK; ^6^ Freie Universität Berlin Fachbereich Biologie, Chemie und Pharmazie Takustraße 3 14195 Berlin Germany

**Keywords:** EPR spectroscopy, fibrous proteins, metabolism, NMR spectroscopy, radicals

## Abstract

Alkaptonuria (AKU) is a rare disease characterized by high levels of homogentisic acid (HGA); patients suffer from tissue ochronosis: dark brown pigmentation, especially of joint cartilage, leading to severe early osteoarthropathy. No molecular mechanism links elevated HGA to ochronosis; the pigment's chemical identity is still not known, nor how it induces joint cartilage degradation. Here we give key insight on HGA‐derived pigment composition and collagen disruption in AKU cartilage. Synthetic pigment and pigmented human cartilage tissue both showed hydroquinone‐resembling NMR signals. EPR spectroscopy showed that the synthetic pigment contains radicals. Moreover, we observed intrastrand disruption of collagen triple helix in pigmented AKU human cartilage, and in cartilage from patients with osteoarthritis. We propose that collagen degradation can occur via transient glycyl radicals, the formation of which is enhanced in AKU due to the redox environment generated by pigmentation.

## Graphical abstract

**Alkaptonuria** is a rare disease that leads to striking pigmentation and mechanical failure of cartilage tissue. DNP‐enhanced solid‐state NMR enabled us to observe disruption of interstrand hydrogen bonding in collagen proteins from alkaptonuria patient tissue. A mechanism involving transient glycyl radicals is proposed as basis of the degradation and pigmentation of cartilage tissue in alkaptonuria.

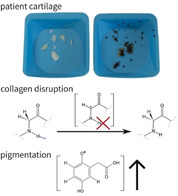

## Introduction

Alkaptonuria is one of the earliest known diseases resulting from an “inborn error of metabolism” as described by Archibald Garrod in 1909. It is caused by mutations in the *HGD* gene^1^ of a key metabolic enzyme, homogentisate 1,2‐dioxygenase, responsible for the breakdown of homogentisic acid (HGA, structure in Supporting Information Figure S1). HGA is released from the liver during tyrosine catabolism^2^ and circulates at high levels in AKU patients. HGA is hypothesized to oxidize to BQA (Figure S1), eventually leading to ochronosis: a dark brown pigmentation in joint cartilage, heart valves, and spinal discs.^3^, ^4^ Ochronosis stiffens cartilage and leads to a highly debilitating osteoarthropathy, especially of weight‐bearing joints,^5^ where patients have little recourse apart from palliative analgesia and complete surgical replacement.

Nitisinone lowers HGA levels by reversibly inhibiting the HGA‐producing enzyme,^6^ and can arrest ochronosis^7^ though not reverse^8^ it. Unfortunately, the FDA considers HGA elevation a biomarker but not a cause of ochronosis,^9^ therefore, nitisinone is thus far not approved for US patients. There is a need to conclusively establish a link from HGA elevation to the cartilage degradation seen in ochronosis.

Solid‐state NMR (ssNMR) enables structural characterization of pigmented tissue biopsies. The hypothesized pigmentation species HGA and BQA are distinguishable by their characteristic chemical shifts (Table S2). Previous ssNMR studies on AKU cartilage only indicated a global broadening of the NMR signals.^10^ With recent developments in dynamic nuclear polarization (DNP) ssNMR,^11^ whereby the observed signal can be enhanced by factors of one to two orders of magnitude, it is attractive to revisit the ssNMR approach for studying AKU tissue.

DNP‐enhanced ssNMR has been successfully applied to non‐isotope enriched (unlabelled) organic materials^12^ and biological materials^13^ to yield informative 2D spectra. It has also enabled ^1^H–^13^C heteronuclear correlation (HETCOR) experiments on heterogeneous biomaterials such as bone^14^ and maize stems.^15^ Here, we obtained 2D spectra of human cartilage samples and gained insight into the structural change in collagen proteins upon pigmentation in AKU.

Since the most debilitating symptom of AKU is a chronic and accelerated form of osteoarthritis (OA), we also investigated structural changes occurring in OA cartilage matrix. This comparison was designed to test the hypothesis that AKU is an extreme form of OA in which similar molecular changes occur, and propose a new mechanism for age‐related cartilage degeneration that can lead to common osteoarthritis.

## Results

We investigated knee cartilage tissues from patients with AKU and OA. All human tissues were formalin‐fixed in order to reduce possible hazards of transmissible diseases.

The 1D ^13^C CP ssNMR spectra of human cartilage showed DNP enhancement values (*ϵ*_ON/OFF_) of 22.5–34.5 under DNP (Table S1). Accounting for depolarization,^16^ we conservatively estimate a time‐saving of one to two orders of magnitude. The effects of formalin‐fixation were investigated (Figure S2) and found to not interfere with HGA‐derived pigment signals.

**Figure 1:**
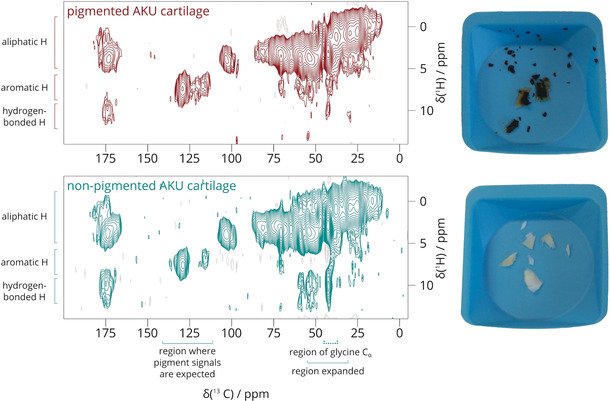
2D ^1^H–^13^C HETCOR DNP‐enhanced ssNMR spectrum of pigmented (red) and non‐pigmented (green) human AKU cartilage at 50 μs contact time. The ^13^C NMR range of 110–140 ppm, where pigment signals are expected, is expanded in Figure 2 A. The 30–55 ppm region containing the glycine C_α_^13^C signal (42.5 ppm) is expanded in Figure 3.

**Figure 2:**
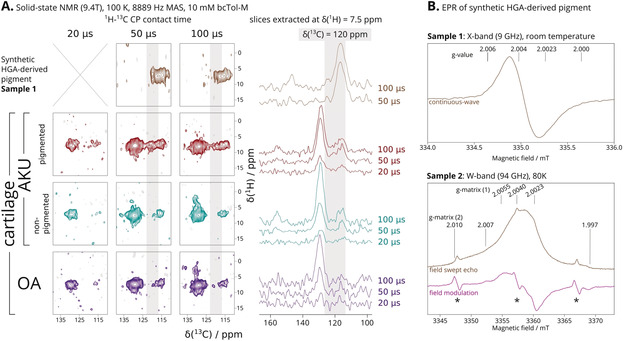
A) ^13^C aromatic region 110–140 ppm sections of ssNMR ^1^H–^13^C HETCOR spectra of: synthetic HGA‐derived pigment (brown, top row), pigmented (red, second row) and non‐pigmented cartilage (green, third row) from AKU patient, and cartilage (purple, bottom row) from OA patient. DNP enhancement was not obtained on the synthetic pigment. Grey stripes highlight the signals attributed to the AKU pigment, also in extracted slices (right) which we also present as an overlay (50 μs, Figure S5). B) EPR spectra of two aqueous solutions of the synthetic HGA‐derived pigment. Sample 1 was recovered from the solid‐state NMR rotor on which DNP enhancement was attempted, on which a room‐temperature X‐band EPR continuous‐wave spectrum was acquired. Sample 2 did not have other radical species added. Here, a W‐band EPR field swept echo spectrum (brown) was acquired at 80 K, and the pseudo field modulation spectrum (magenta) calculated. Two components with different g‐matrices, (1) narrow and (2) broad, can be distinguished. Asterisks (*) indicate Mn^2+^ impurities.

To identify the pigment NMR signals, we compared three types of cartilage: heavily pigmented cartilage and visually non‐pigmented cartilage from the same AKU patient, and cartilage from a patient with OA. The DNP‐enhanced ^1^H–^13^C HETCOR 2D spectra of pigmented and non‐pigmented human AKU cartilage are shown in Figure 1. As expected, both spectra are dominated by collagen signals overall, but differences between the spectra of pigmented and non‐pigmented AKU cartilage are apparent in the ^13^C aromatic region (110–140 ppm), expanded in Figure 2 A.

**Figure 3:**
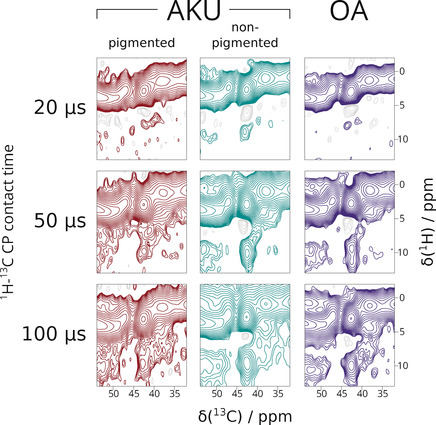
^13^C aromatic region 30–55 ppm of DNP‐ssNMR ^1^H–^13^C HETCOR spectra of pigmented AKU human cartilage (red, left column), non‐pigmented AKU human cartilage (green, middle column) and OA human cartilage (purple, right column). An overlay of the extracted slices (100 μs) is provided in Figure S7.

To detect the presence of HGA and its derivatives, we recorded a series of HETCOR experiments with different CP contact times (Figure 2 A and Figure 3). Signals from more distal ^1^H and ^13^C pairs emerge with increasing CP contact time.

In Figure 2 A, the HETCOR spectra of all cartilage samples had a broad ^13^C signal at 130 ppm corresponding to aromatic residues (tyrosine, phenylalanine, histidine) and a narrower signal at 115 ppm corresponding to tyrosine. In pigmented cartilage (Figure 2 A, second row, red), there is an additional signal at 120 ppm that is absent in the non‐pigmented AKU cartilage and the OA cartilage. This signal coincides with the spectrum of the synthetic HGA‐derived pigment (Figure 2 A, top row, brown). Thus, we assigned the 120 ppm signal in pigmented cartilage to aromatic carbons in the HGA‐derived pigment.

The 120 ppm NMR signal strongly indicates a hydroquinone‐like functionality in the pigment. Hydroquinones are known for their redox activity. Therefore, the HGA‐derived pigment was investigated by EPR spectroscopy (Figure 2 B), which confirmed the presence of an air‐ and water‐stable radical. The signal is narrow, without resolved hyperfine coupling, and relatively easy to saturate even at low microwave power. At least two distinct radical species are present within the HGA‐derived pigment, suggesting some reactivity.

After detecting the pigment by NMR and EPR, we considered the effects of pigmentation on collagen structure. We inspected the spectra and found reproducible changes in the glycine C_α_ signals, which we consider as monitors of collagen structural integrity. In NMR spectra of collagenous tissues such as cartilage, this signal is one of the most intense since glycines occur every third residue along each collagen chain. These signals combine into one ^13^C peak centered at 42.7 ppm, well separated from C_α_ signals of other amino acids. In Figure 3, we expand this particular spectral region from the HETCOR spectra.

At short contact times (top row), the glycine C_α_ signal at 42.7 ppm mainly correlates to an agglomerate of ^1^H signals centered at 3.3 ppm, which correspond to the directly bonded glycine H_α_s (1.1 Å). As we increased contact time, a second correlation emerges, centered at 9.6 ppm (^1^H) in the spectra of the non‐pigmented AKU cartilage (green, middle column) and the OA cartilage (purple, right column). We attribute this signal to correlations between glycine C_α_ and the respective H_N_ (≈2.1 Å). The high ^1^H chemical shift value of 9.6 ppm is due to the interchain hydrogen bonding that is a defining feature of all collagen triple helices.

In the pigmented cartilage spectra (Figure 3, left column/red), the glycine C_α_‐H_N_ signal is not as well‐defined and smeared towards lower ^1^H chemical shift values, which can also be seen in the ^1^H columns extracted at *δ*(^13^C)=42.5 ppm (Figure S7). This indicates that the hydrogen bonds that the glycine H_N_ are participating in are disrupted, suggesting partial dissociation of the collagen triple helix in the pigmented AKU cartilage sample.

To achieve better spectral resolution in the glycine region, we conducted room temperature HETCOR experiments. Although standard ssNMR acquired over a much longer experiment time (47 hours instead of 10 hours per experiment) at 287 K on a 600 MHz spectrometer did not show clear resolution improvement (Figure S9), we were able to confirm our DNP results in that a glycine C_α_‐amide H_N_ signal can be observed in non‐pigmented cartilage, but is absent from pigmented cartilage.

## Discussion

In this work, we showed that NMR structural investigations can be applied to formalin‐fixed samples, complementing histological approaches, magnetic resonance imaging,^17^ and mass spectrometry.^18^ With DNP enhancement of natural abundance human samples, we observed a ^13^C signal at 120 ppm that we ascribe to AKU pigmentation.

We surveyed the NMR literature for likely chemical structures of the pigment. The broad ^13^C signals centered at 116.8 ppm and 148.5 ppm from the HGA‐derived pigment sample are characteristic of phenol ring carbons, in agreement with hydroquinone‐ or HGA‐like ring structures.^19^, ^20^ If benzoquinone rings are present, we would expect ^13^C signals at 136 ppm, which we clearly did not observe (Figure 2 A).

As BQA‐related NMR signals were not observed, the mechanism by which pigmentation proceeds in AKU should be reassessed. A new hypothesis involves the formation of a radical derived from HGA. Hydroquinones are known for their redox properties and play important roles in a range of biological processes,^21^ where the formation of a radical semiquinone is key to the transformation between the fully oxidized quinone and reduced quinol species. X‐band EPR spectra (Figure 2 B) of the HGA‐derived radical resembled that of melanins^22^ and polyhydroquinones,^23^ while the spectrally narrower component (1) in W‐band EPR spectra resembled semiquinone radical anions.^24^ Keeping in mind that similarity in electronic structure as measured by EPR does not require high similarity in chemical structure, we refer back to NMR literature in order to distinguish chemical structure differences in HGA pigment and melanins.

The AKU pigment has been compared to hair melanin and bacterial pyomelanin.^25^ The brown HGA‐derived pigment had a relatively simple ^13^C ssNMR spectrum dominated by four signals (Figure S2 C). By comparing the NMR spectra in the current study with previous ssNMR work on melanin in fungal cell wall,^26^ hair samples,^27^ and squid ink,^28^ we confirm that the structure of the pigment is different from l‐dopa‐based melanins, but is consistent with a hydroquinone functionality. We suspect more similarities will be found with bacterial pyomelanins, but unfortunately NMR study of this material is not yet available.

We therefore propose the oxidation of HGA to the corresponding semiquinone radical as the first step of the pigmentation process. This enables us to neatly explain several aspects with relevance to AKU:

- The reactivity of radical species in cartilage tissue can lead to changes in cartilage material properties (vide infra) seen in patients with AKU.
- Radicals are known to exhibit distinct and intense colours; electron delocalization can redshift the absorption spectrum.
- The persistence of hydroquinone moieties in the pigment, both in the long‐known (but unexplained) infrared spectra of brown solutions of oxidized HGA^29^ and our NMR work here.
- The low sensitivity of pigment NMR signals, since radicals can broaden or even suppress signals by enhancing relaxation.
- Presence of a reducing substance in AKU patients’ urine was already reported in 1859.^30^
- Presence of radicals in samples with elevated HGA concentration can explain the observed negative interference on routine clinical assays that are based on the production of hydrogen peroxide.^31^

With these aspects in mind, we envisage new clinical assays for detecting HGA pigmentation and AKU disease progression via EPR spectroscopy.

After establishing the importance of radical species in HGA‐derived pigmentation, we consider processes by which pigmentation can affect the structure of collagen. The ^1^H‐^13^C HETCOR spectra showed remarkable changes in triple helix integrity between non‐pigmented and pigmented AKU cartilage. The observation that the ^1^H chemical shift of glycine amide moieties shows heterogeneous lower values indicate lengthening and weakening of these hydrogen bonds.^32^, ^33^ In collagen, where these interchain hydrogen bonds are pervasive, such structural change will likely lead to mechanical and functional failures of pigmented cartilage.

We propose that transient formation of glycyl radicals underlie the disruption of hydrogen bonding in pigmented cartilage observed in the current study. Molecular dynamics indicate that the formation of a glycyl radical is accompanied by large changes in the backbone dihedral angles,^34^ since hydrogen abstraction occurs at C_α_ of glycines,^35^ and have been observed in EPR of powdered collagen (freeze‐dried and rehydrated) subjected to gamma rays.^36^ While glycyl radicals are known to be key to many enzymatic processes, they are not usually oxygen‐stable.^37^, ^38^, ^39^ However, in this case, stability of the glycyl radical is not necessary; even transient radical formation would lead to disruption of intrastrand hydrogen bonding in collagen, which was observed via DNP NMR, indicating local unfolding of the collagen triple helix that may be made irreversible by covalent modification of the radical sites.

Collagen type II, the most abundant structural component of cartilage, shows minimal^40^ to very slow turnover (half‐life 6.6 years).^41^ Over time, transient glycyl radicals form in AKU cartilage, enabled by initial, low‐level HGA radical formation. Once formed, such glycyl radicals can oxidize nearby HGA molecules, and perpetuate the process, whereby increased oxidation of HGA in cartilage, further pigment formation, and further collagen damage can occur.

The glycyl radical hypothesis can explain the relatively sudden onset^42^ of ochronotic osteoarthropathy and non‐uniform distribution of ochronosis throughout the body. Mechanical load on collagen^43^ and bone fracture^44^ generate radicals, and may also predispose formation of glycyl radicals, thus underlie the observation that load‐bearing parts of cartilage are first to pigment in AKU. The initial kinetic barrier for glycyl radical formation explains why cartilage at different sites acquire pigmentation at different rates. The elevated HGA levels provide an environment that is especially susceptible to further oxidative stress, ultimately resulting in ochronosis and loss of cartilage function.

In AKU, osteoarthropathy is predictable and inevitable, hence the interest in studying the disease as a model of common OA. Our ssNMR results enabled us to make direct comparisons of the glycine H_N_ hydrogen bonding in AKU and OA cartilage. Although the spectra obtained from OA cartilage is broadly similar to that obtained from non‐pigmented AKU cartilage, the most intense part of the glycine H_N_ signal drifted to lower chemical shift. Our interpretation is that OA cartilage showed an intermediate level of collagen triple helix degradation; more than non‐pigmented AKU cartilage and less than pigmented AKU cartilage, and that glycyl radical formation can also be a relevant mechanism in OA. Our work supports the idea that AKU is an extreme form of common OA which can be used to model the earliest age‐related degenerative changes to joint cartilage.

## Conflict of interest

The authors declare no conflict of interest.

## Supporting information

As a service to our authors and readers, this journal provides supporting information supplied by the authors. Such materials are peer reviewed and may be re‐organized for online delivery, but are not copy‐edited or typeset. Technical support issues arising from supporting information (other than missing files) should be addressed to the authors.

Supplementary
