# Supplementary material for: Pigmentation Chemistry and Radical‐Based Collagen Degradation in Alkaptonuria and Osteoarthritic Cartilage
Source: Angew Chem Int Ed Engl. 2020 May 14;59(29):11937–42. doi: 10.1002/anie.202000618 (PMC7383862; doi:10.1002/anie.202000618)
Supplement: Supplementary file 1 — Supplementary [file ANIE-59-11937-s001.pdf]

Angewandte Chemie
Eine Zeitschrift der Gesellschaft Deutscher Chemiker

Supporting Information

Pigmentation Chemistry and Radical-Based Collagen Degradation in Alkaptonuria and Osteoarthritic Cartilage**

Wing Ying Chow, Brendan P. Norman, Norman B. Roberts, Lakshminarayan R. Ranganath, Christian Teutloff, Robert Bittl, Melinda J. Duer, James A. Gallagher,* and Hartmut Oschkinat**

anie_202000618_sm_miscellaneous_information.pdf

Author Contributions

W.C. Conceptualization: Lead; Data curation: Lead; Formal analysis: Equal; Funding acquisition: Supporting; Investigation: Equal; Methodology: Lead; Project administration: Lead; Visualization: Lead; Writing - Original Draft: Equal; Writing - Review & Editing: Equal
B.N. Conceptualization: Supporting; Data curation: Supporting; Funding acquisition: Supporting; Investigation: Equal; Methodology: Equal; Project administration: Supporting; Resources: Equal; Validation: Supporting; Writing - Original Draft: Equal; Writing - Review & Editing: Equal
N.R. Funding acquisition: Supporting; Methodology: Supporting; Resources: Supporting; Supervision: Supporting; Validation: Equal; Writing - Review & Editing: Supporting
L.R. Formal analysis: Supporting; Funding acquisition: Equal; Resources: Supporting; Writing - Review & Editing: Supporting
C.T. Formal analysis: Supporting; Investigation: Supporting; Methodology: Supporting; Resources: Supporting; Visualization: Supporting; Writing - Review & Editing: Supporting
R.B. Resources: Supporting; Supervision: Supporting; Validation: Supporting; Writing - Review & Editing: Supporting
M.D. Validation: Equal; Writing - Original Draft: Equal; Writing - Review & Editing: Supporting
J.G. Conceptualization: Equal; Funding acquisition: Lead; Methodology: Equal; Project administration: Equal; Resources: Equal; Supervision: Equal; Validation: Equal; Writing - Original Draft: Equal; Writing - Review & Editing: Supporting
H.O. Formal analysis: Equal; Funding acquisition: Equal; Methodology: Supporting; Resources: Lead; Supervision: Equal; Validation: Equal; Visualization: Equal; Writing - Original Draft: Supporting; Writing - Review & Editing: Equal.

Abstract
Alkaptonuria (AKU) is a rare disease characterized by high levels of homogentisic acid (HGA); patients suffer from tissue ochronosis: dark brown pigmentation, especially of joint cartilage, leading to severe early osteoarthropathy. No molecular mechanism links elevated HGA to ochronosis; the pigment's chemical identity is still not known, nor how it induces joint cartilage degradation. Here we give key insight on HGA-derived pigment composition and collagen disruption in AKU cartilage. Synthetic pigment and pigmented human cartilage tissue both showed hydroquinone-resembling NMR signals. EPR spectroscopy showed that the synthetic pigment contains radicals. Moreover, we observed intrastrand disruption of collagen triple helix in pigmented AKU human cartilage, and in cartilage from patients with osteoarthritis. We propose that collagen degradation can occur via transient glycyl radicals, the formation of which is enhanced in AKU due to the redox environment generated by pigmentation.

Supporting Information

Table of Contents

- Experimental Procedures - page 3
    - Human cartilage tissue collection
    - WT and HGD(-/-) mouse tissue collection
    - Tissue fixation, shipping, and preparation for NMR experiments
    - HGA-derived pigment formation
    - ssNMR rotor packing
    - EPR spectroscopy
    - Solid-state NMR spectroscopy
    - NMR experiment details on determination of DNP enhancement value
    - NMR spectra processing
- Results and Discussion (Supporting) - page 7
    - Current hypothesis of pigmentation
    - Supplementary Table S1: Samples investigated in this study and enhancement values
    - Effects of formalin fixation
    - Supplementary Figure S2: 13C 1D spectra of cartilage samples used to investigate the effect of fixation
    - Supplementary Table S2: Experimental and expected 13C NMR chemical shift of the structures relevant to AKU pigmentation
    - Supplementary Table S3: Human cartilage sample details
    - Supplementary Table S4: Mouse condyle sample details
    - Supplementary Figure S5: 13C spectra extracted from the 2D ssNMR 1H-13C FSLG HETCOR DNP-enhanced ssNMR spectra of human cartilage
    - Supplementary Figure S6: 1H-13C FSLG HETCOR ssNMR spectra of **unsoaked** human cartilage
    - Supplementary Figure S7: 1H spectra extracted from the 2D ssNMR 1H-13C FSLG HETCOR DNP-enhanced ssNMR spectra of human cartilage
    - Supplementary Figure S8: 1H-13C FSLG HETCOR DNP-enhanced ssNMR spectra of unsoaked human cartilage
    - Supplementary Figure S9: 1H-13C FSLG HETCOR ssNMR spectra of human cartilage
- References - page 19
- Author Contributions - page 20

Experimental Procedures

Human cartilage tissue collection

All cartilage tissue was obtained as surgical waste under ethical approval from the Liverpool Research Ethics Committee (REC) with informed consent from patients with diagnosis of AKU or OA undergoing joint replacement. Human joint tissue was obtained at the time of surgery from the knee of a 55 year old male previously diagnosed with AKU, and of a 71 year old female patient with osteoarthritis. Both patients underwent a complete knee replacement due to osteoarthropathy. For the AKU specimen, macroscopically pigmented and non-pigmented sections of articular cartilage were dissected from the same tissue sample. For the OA specimen, samples were taken from regions of articular cartilage that were visually worn, 'damaged' tissue, and also from regions of articular cartilage that were not visually damaged, referred to as 'distal' tissue. Samples were dissected using a No. 10 disposable scalpel (Swann-Morton, UK) and washed with sterile PBS before storage at -80°C. Further details are provided in Supplementary Table S3.

WT and *HGD*(-/-) mouse tissue collection

Mouse breeding was authorised under UK Home Office guidelines. Mouse tissues were obtained from mice of different genetic backgrounds. The distal condyles of the femur, which are covered by cartilage, was taken from $HGD^{-/-}$ and wild type mice using a No. 10 disposable scalpel (Swann-Morton, UK). Due to the small size of the condyles, we did not attempt to separate the cartilage from the underlying bone. Cartilage of the $HGD^{-/-}$ mice are microscopically pigmented, while cartilage of wild type mice are not pigmented. Tissue from BALB/c and C57BL/6J mouse strains were combined in both of the above groups to give sufficient material for rotor packing. Samples were washed in sterile PBS and stored at -80°C unless intended for subsequent fixation. Further details are provided in Supplementary Table S4.

Tissue fixation, shipping, and preparation for NMR experiments

Mouse tissue samples designated for fixation were fixed immediately following dissection. Human tissues were removed from -80°C storage and thawed prior to fixation.

Tissues were soaked in twice the tissue volume of 10% formalin (pH 7.4) for 48 hours with gentle agitation. The formalin was changed after 24 hours. After 48 hours, tissues were removed from the formalin and stored short-term at 4°C in tubes containing paper towel soaked in formalin to prevent drying.

The fixed samples (mouse and human) were shipped at ambient conditions while the non-fixed mouse tissue samples were shipped on ice to the NMR facility. All tissues were stored at -20°C prior to NMR experiments.

In order to remove the large NMR signal from the formalin fixative, some of the fixed human cartilage was washed by rinsing in distilled water, then soaked in distilled water with one change of water for up to one week prior to the NMR experiments.

Data for samples used directly without soaking to remove the fixative was presented in Supplementary Fig. S6 and Supplementary Fig. S8.

HGA-derived pigment formation

10 mL of a 10 mM aqueous solution of HGA was incubated in a glass vial under aerobic conditions at 37°C. Upon the addition of 30 μL of a 5M NaOH solution to raise the pH, an immediate darkening of the solution was observed, which progressed over the total incubation period of approximately 4 months to yield a brown solution with a small amount of sedimentation (Supplementary Fig. S4A).

About 2 mL of the HGA-derived pigment solution was dried to produce a solid sample for ssNMR experiments. A brittle, dark brown solid was obtained after drying under a stream of dry nitrogen gas. During the drying process, it was noted that the pigment is highly soluble in water. The pigment is easily solubilized in water to give a dark brown solution (Supplementary Fig. S4B).

ssNMR rotor packing
No grinding or cryomilling procedures were used on the biological samples. The mouse condyles were analysed without further dissection, while the cartilage samples were cut into smaller pieces prior to rotor filling. A photo of the cartilage samples prior to rotor packing is provided in Supplementary Fig. S3. The samples were gently dried by a combination of two techniques: brief blotting using a paper towel, or pressed briefly between two layers of Parafilm to expel water, which was then removed with a fine pipette. No freeze drying was used. For all NMR experiments, 3.2 mm outer diameter zirconia rotors with Vespel caps were used. The small pieces of human cartilage were placed directly into the rotor with slight compaction if necessary. For the mouse tissue, condyles from different mice had to be pooled to provide sufficient sample mass.

Due to the difficulty of transferring small amounts of the HGA-derived brown solid into the rotor, the solid was dissolved in a small amount of water. The resulting brown solution was then transferred to the rotor, and a stream of dry nitrogen gas was used to remove the water. For DNP experiments, AMUPol[45], bcTol[46], and bcTol-M[47] were prepared as solutions in D_2O only, to a concentration of 20 mM for AMUPol and 40 mM for bcTol and bcTol-M. A range of radicals was used due to development of new and improved radicals over the course of the project. Depending on the tissue mass, 5-7 µL of the radical solution was added to the filled rotor. No glycerol was used as it was previously shown to bind to native collagen and modify its stability[48]. No H_2O as added, as the samples were well-hydrated and we expect a sufficient amount of H_2O to remain even after the gentle drying process. The procedure was based on the incipient wetness impregnation technique that is used widely in DNP NMR studies of materials[49].

After adding the radical solution, the packed rotors were centrifuged to distribute the radical solution throughout the sample, and then left to equilibrate overnight at 4 °C before the start of DNP experiments.

For the HGA-derived pigment sample, 5 µL of 40 mM bcTol radical solution (in D_2O) was added to the dried pigment in the rotor. After the initial unsuccessful DNP attempt, the rotor was opened and a further 5 µL of 40 mM bcTol radical solution (in D_2O) added, with the assumption that insufficient radical was added initially.

EPR spectroscopy
For all EPR experiments, the sample preparation and experiment was carried out on the open bench without excluding oxygen. In all experiments, the magnetic field was calibrated using a N@C60 g-standard[50].

X-band (9 GHz): Continuous wave (cw) EPR spectra were recorded at room temperature on a home built X-band spectrometer featuring a Bruker ER 041 MR microwave source, a Bruker ER 4122-SHQ E resonator optimized for cw-measurements, a Stanford Research Systems SR810 lock-in amplifier, an AEG electromagnet, driven and controlled by a Bruker ER 081S power supply and a Bruker B-H 15 field controller. Spectra were recorded at a microwave power of 45 dB with 1 G field modulation and a time constant of 100 ms. The sample was prepared in quartz tubes of 2/0.8 mm (outer/inner diameter) (Quarzschmelze Ilmenau). A dark brown, aqueous solution (D_2O/H_2O ~ 1:5) of HGA-derived pigment was recovered from the solid-state NMR rotor and used for the X-band EPR experiment.

W-band (94 GHz): Cryogenic temperature (80K) EPR measurements were performed using a W-band EPR spectrometer (Bruker Elexsys E680 S/W-Band) equipped with a Teraflex EN600-1021H probe head (Bruker Biospin, Karlsruhe, Germany). The temperature was controlled by an Oxford ITC 503 temperature controller. The field-swept echo (fse) spectra were conducted in a Hahn-echo fashion (π/2-tau-π-τ-echo) with a π/2 pulse length of 40 ns and a pulse separation time of 300 ns. The cw-like spectrum was calculated from the fse spectrum by pseudo-modulation (modulation amplitude 1 mT). The sample was prepared in quartz tubes of 0.87/0.7 mm (outer /inner diameter) (VitroCom Inc, Mountain Lakes, USA). A light brown, aqueous solution (H_2O) of HGA-derived pigment that was never used for DNP ssNMR experiments was used for the W-band EPR experiment.

Solid-state NMR spectroscopy

NMR experiments were performed on Bruker Avance III wide-bore spectrometers operating at static magnetic fields of 9.4 T and 14 T, with ^{1}H Larmor frequencies of 400 and 600 MHz respectively. The 600 MHz spectrometer was equipped with a 3.2 mm (^{1}H-^{13}C-^{15}N) triple resonance probe, with the magic angle spinning (MAS) rate set to 13333 Hz. The sample temperature was maintained using a Bruker cooling unit (BCU) with a gas flow of 1200 litres per hour. The sample temperature was set to 265 K; after calibration, the actual temperature was found to be 285 K. The 400 MHz spectrometer is equipped for DNP enhancement experiments, with a gyrotron operating at 9.7 T and a 3.2 mm (^{1}H-^{13}C-^{15}N) triple resonance low temperature magic angle spinning (LTMAS) probe. The MAS rate was set to 8889 Hz, and the sample temperature set to 100 K (actual temperature estimated to be 110 K) using the LTMAS cooling cabinet with a variable temperature gas flow of 2000 litres per hour.

NMR parameters on the 400 MHz DNP spectrometer were: ^{1}H 90° pulse length 2.5 μs, ^{13}C 90° pulse length 3.8 μs, 88 kHz ^{1}H decoupling. DNP-enhanced experiments were carried out under 20-30 mA microwave irradiation. NMR parameters on the 600 MHz spectrometer were: ^{1}H 90° pulse length 3.25 μs, ^{13}C 90º pulse length 5 μs, 73 kHz ^{1}H decoupling.

^{1}H-^{13}C cross polarisation (CP) experiments are used to improve sensitivity in ssNMR. It is based on dipolar coupling and therefore specifically detects immobile and solid species. 600 μs ^{1}H-^{13}C CP contact time was used on the 400 MHz DNP spectrometer. 1000 μs ^{1}H-^{13}C CP contact time was used on the 600 MHz spectrometer. A ramped shape was used on the carbon channel during the CP contact to broaden the matching condition[51].

^{1}H-^{13}C refocused insensitive nuclei enhanced by polarisation transfer (INEPT)[52] experiments are a common technique for spectral editing in ssNMR, specifically for detecting mobile and soluble species as the transfer is based on J-coupling, which is not averaged to zero under MAS, and the relatively long delays lead to broadening of solid/immobile signals from T_2 relaxation. This experiment was only carried out on the 600 MHz spectrometer at 285 K and not carried out at DNP conditions. The INEPT delay was 1.5 ms and the refocusing delay was 0.9 ms.

^{1}H-^{13}C 2D heteronuclear correlation (HETCOR) spectra were carried out with a frequency-switched Lee-Goldburg (FSLG) sequence during ^{1}H evolution[53] to improve resolution, with a square shape applied on both ^{1}H and ^{13}C during the CP contact step. At the 400 MHz spectrometer, under DNP conditions, CP contact times of 20–100 μs were recorded for several samples, with some samples additional contact times of 10 and 500 μs were also recorded. Each HETCOR experiment was signal averaged for 5-10 hours. On the 600 MHz spectrometer, for each human cartilage sample, two experiments with contact times of 500 μs and 1000 μs were performed, with each experiment taking 27-47 hours.

NMR experiment details on determination of DNP enhancement value

256 transients were acquired per 1D spectrum for human samples, while 3072 transients were acquired for mouse samples. The range of radicals used, including AMUPol[45],

bcTol[46], and bcTol-M[47] was due to improved radicals being available through the course of the study.

NMR spectra processing

For 1D ^{13}C spectra, 1024 points of the experimental FID (12.5 ms) was zero filled to 8192 points. A Gaussian window function was applied, with 10 Hz line broadening and a Gaussian max position (GB in Bruker Topspin) of 0.1, prior to Fourier transform. Baseline correction was applied to the whole spectral range of -100 to 300 ppm. ^{13}C spectra were externally referenced to tetramethylsilane (TMS) to enable comparison to the chemical literature of polyquinones. However, this referencing method leads to a small (2.7 ppm) discrepancy of the chemical shift values of any protein signals from those found in the literature of other protein NMR studies, which are often referenced to 2,2-dimethylsilapentane-5-sulfonic acid (DSS). Previous collagen NMR chemical shifts were reported by referencing to TMS.

For 2D ^{1}H-^{13}C spectra, 1024 points of the direct dimension (^{13}C, 12.5 ms) was zero filled to 8192 points, and 96 points of the indirect dimension (^{1}H, 5 ms) was zero filled to 512 points. As is common for FSLG experiments, the ^{1}H dimension scaling factor is empirically determined. A Gaussian window function was applied to the direct dimension, with 30 kHz line broadening and a Gaussian max position (GB in Bruker Topspin) of 0.1. For the indirect dimension, two processing regimes were used: for plots of the carbon aromatic region, a Gaussian window function was applied with 20 Hz line broadening and a Gaussian max position of 0.1; for plots of the glycine C^{α} region, a sine bell squared function was applied with a sine bell shift of 4. Baseline correction was applied to the full spectral range.

Results and Discussion (Supporting)

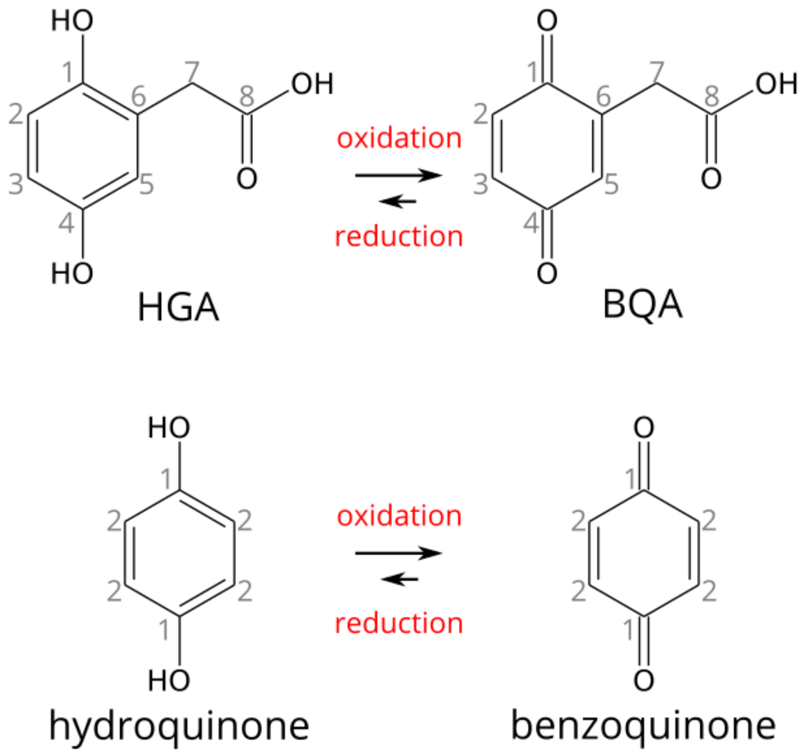

Supplementary Figure S1: Chemical structures of HGA (homogentisic acid, IUPAC name 2,5-dihydroxyphenylacetic acid), BQA (benzoquinone acetic acid), hydroquinone and benzoquinone. The grey annotations indicate carbon atoms that would give rise to distinct NMR signals, selected to give a consistent labelling scheme throughout this manuscript. Note that the numbering is different from that used in the IUPAC name of HGA. In the case of hydroquinone and benzoquinone, many carbons are symmetry-related, meaning that a much smaller range of carbon signals is expected.

Current hypothesis of pigmentation

HGA is water-soluble and should be easily removed from body tissues by circulation. While HGA is colourless, it becomes dark brown upon oxidation, ostensibly to benzoquinone acetic acid (BQA) (Supplementary Fig. S1). In oxidized HGA solutions, which can be produced non-enzymatically at physiological pH,[54] the presence of a separable brown component, thus far attributed to BQA, has been shown by chromatography[55] and UV-Vis spectroscopy.[56] As HGA is present at high levels in the urine of patients with AKU,[57] the dark colour of the urine upon oxidation was attributed to BQA formation. BQA is thought to underlie the visually striking discolouration (Supplementary Fig. S3) and accompanying tissue degradation in ochronotic joint cartilage.

In the current model of pigmentation, HGA is first oxidized to BQA, then undergoes aggregation or polymerization to yield a final pigment species[25] containing benzoquinone moieties (Supplementary Fig. S1), giving rise to the dark colour. Alkaline air oxidation of colourless HGA solutions generate a brown solution (Supplementary Fig. S4), and a dark brown, flaky powder can be obtained with subsequent freeze drying,[58] but NMR study of the chemical products of this reaction are lacking. Using infrared spectroscopy, it was shown that oxidized (coloured) and fresh (colourless) HGA solutions are highly similar,[29] which strongly suggests that the conversion of HGA to BQA (if any) is highly incomplete, and that the hydroquinone functionality remains substantial even in coloured solutions of oxidized HGA. A question remains on how similar the synthetic HGA-derived colourant is to the pigment observed in AKU patients, due to the difficulty of isolating the pigment from patient samples.

Supplementary Table S1: Samples investigated in this study and enhancement values (microwave on/off intensity ratio) obtained from CP-MAS ssNMR ^{13}C 1D spectra. The complete lack of DNP enhancement of the AKU-derived pigment also prompted us to hypothesize that the pigment was capable of quenching the added binitroxide radical.

Sample source	Tissue	Treatment	Radical	Enhancement
Human AKU patient	Non-pigmented articular cartilage	Formalin fixation	bcTol	22.5 ± 1.8
Human AKU patient	Pigmented articular cartilage	Formalin fixation	bcTol	35.4 ± 5.4
Human OA patient	Osteoarthritic articular cartilage	Formalin fixation	bcTol-M	25.5 ± 3.5
Mouse WT (unpigmented)	Femur condyles	No treatment	bcTol	12.5 ± 5.2
Mouse Hgd (pigmented)	Femur condyles	No treatment	AMUPol	14.3 ± 5.3
Mouse WT (unpigmented)	Femur condyles	Formalin fixation	bcTol	20.3 ± 12.3
Mouse Hgd (pigmented)	Femur condyles	Formalin fixation	AMUPol	16.1 ± 4.5
HGA-derived pigment	-	Drying	bcTol	0

Effects of formalin fixation

Though previous reports indicate that protein secondary structure is not affected by formalin fixation[59], it may confound NMR spectra changes due to pigmentation. Therefore, a comparison of mouse tissue from wild type and *HGD*$^{-/-}$ mice (as a model for AKU)[60], with and without fixation, was first carried out, in order to distinguish the effects of fixation on NMR spectra from any effects of cartilage pigmentation.

The main effect of fixation on ^{13}C NMR spectra acquired under DNP conditions is a large signal at 82.2 ppm, and a smaller signal at 84.5 ppm. These signals (Supplementary Fig. S2A, yellow boxes) occur in the 70-100 ppm region, indicating formation of methylene glycol and polyoxymethylene glycol from formaldehyde under aqueous conditions[61]. The signals from residual methylene glycol can be eliminated by soaking in distilled water (Supplementary Fig. S2B), and importantly, these signals did not interfere with signals of the synthetic HGA-derived pigment. The ssNMR ^{13}C signals of the HGA-derived pigment (Supplementary Fig. S2C, Supplementary Fig. S4) are well clear of any methylene glycol signals that arise from adsorbed fixative. Therefore, the fixation procedure should not hinder our aims to observe HGA-derived pigment signals in this case.

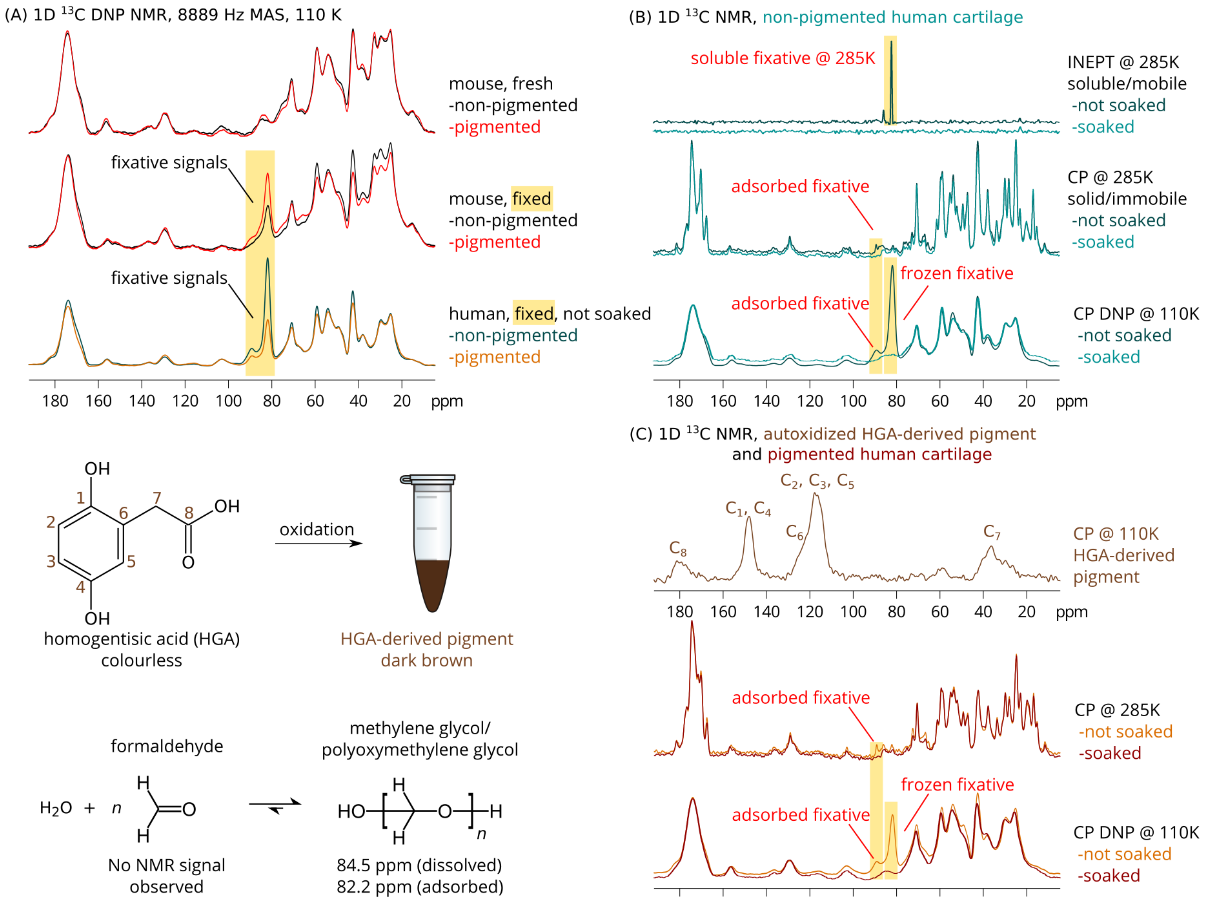

Supplementary Figure S2: ^{13}C 1D spectra of cartilage samples used to investigate the effect of fixation. Signals arising from the fixative are highlighted by yellow boxes. (A) DNP-enhanced NMR spectra of fresh/unfixed mouse tissue (top), fixed mouse tissue (middle) and fixed human tissue (bottom). Black spectra indicates non-pigmented tissue, while red spectra indicates pigmented tissue in the case of the patient cartilage, or microscopically pigmented in the case of the mouse tissue. Bottom inset shows the reaction of formaldehyde with water to produce glycols. (B) Non-pigmented human cartilage NMR INEPT (top two) and CP (bottom four) spectra at 285K (no DNP enhancement, 600 MHz spectrometer) and 110K (with DNP enhancement, 400 MHz spectrometer). Cartilage that were not soaked (dark blue traces) show a signal arising from the fixative, which disappeared after soaking (light blue traces). (C) (top) Autoxidized HGA-derived pigment with each signal assigned to a carbon on the HGA structure. (bottom) Pigmented human cartilage NMR spectra at 285K (no DNP enhancement) and 110K (with DNP enhancement).

From the DNP spectra (Supplementary Fig. S2B, Supplementary Fig. S2C), significant inhomogeneous line broadening clearly occurred under DNP conditions, to the extent that there appears to be little to no difference between the NMR spectra of pigmented and non-pigmented cartilage. The CP spectra of pigmented and non-pigmented cartilage obtained at 285K (Supplementary Fig. S2B, Supplementary Fig. S2C) do show a difference in linewidth as previously demonstrated,[10] and this difference is maintained after soaking both samples. This observation likely indicates a loss of motional averaging and dynamics of the cartilage tissue under DNP conditions.

In the case of the fixed tissues, we have to take into account spectral interference from either the rest of the fixative or the cartilage tissue. We eliminate the fixative as a source of interference since no fixative signals occur in the aromatic region of interest at 110-160 ppm. Of the natural amino acids, we would expect phenylalanine and histidine side chain signals to overlap with those arising from BQA, while tyrosine side chain signals would overlap with HGA. Fortunately, cartilage is predominantly type II collagen, with a low proportion of aromatic residues: phenylalanine (1.4% of the triple helix), histidine (0.2%), tyrosine (0.2%), and no tryptophan. These side chains in the aromatic region should therefore be minor signals in the spectra. Moreover, we have used the non-pigmented AKU cartilage and OA cartilage as controls to establish the expected relative signal intensities arising from these minor aromatic side chains prior to extracting information specific to the pigment species.

Supplementary Table S2: Experimental and expected ^{13}C NMR chemical shift of the structures relevant to AKU pigmentation, as referenced to tetramethylsilane.

Chemical entity	Carbon	Chemical Shift / ppm
HGA-derived pigment in current study	2,3,5	120 (broad)
Homogentisic acid (HGA)[62] *	1,4<br>2,3,5<br>6<br>7<br>8	150.4, 151.6<br>117.2, 119.8, 120.4<br>128.3<br>43.2<br>183.4
Benzoquinone acetic acid (BQA) ^	1,4<br>2,3,5<br>6<br>7<br>8	Expected 187<br>Expected 130-136<br>Expected 136+<br>Expected 40-50<br>Expected 183
Hydroquinone[19] §	1<br>2	151.5<br>118.5
Benzoquinone[19] §	1<br>2	187.0<br>136.4

* Bruker format data of HGA in aqueous solution was downloaded from the Madison Metabolomics Consortium Database (MMCD) and the referencing taken as is. We note that the chemical shift values reported on the HGA page of the MMCD [1] do not seem to match the values from the spectrum downloaded [2], but the chemical shifts of the spectrum downloaded showed reasonable agreement with the solid-state ^{13}C NMR spectrum of the HGA-derived pigment in our study.
[1] http://mmcd.nmrfam.wisc.edu/test/cqsearch.py?cqid=cq_00389
[2] http://mmcd.nmrfam.wisc.edu/rawnmr/expnmr_00219_3.tar
^ Experimental values were not found for BQA, likely due to its instability. Expected chemical shift values were derived with reference to benzoquinone and HGA values in literature.
§ In addition, similar values can be found in the following references: [20,63,64].

The observation of the 187 ppm signal of benzoquinone is made difficult in the case of HGA/BQA due to interference from the acetic acid functionality, which has a ^{13}C chemical shift in the same region. Thus, only the 136 ppm chemical shift is diagnostic of BQA/benzoquinone functionality.

We can rule out lactone formation,[65] which is expected to give a ^{13}C chemical shift less than 178 ppm. The 181 ppm chemical shift that we observe is close to that observed in solution-state NMR at 183.4 ppm (Supplementary Table S2) suggesting that the carboxylate functionality of HGA has not undergone significant change.

Supplementary Table S3: Human cartilage sample details.

<table>
<tr><th>NMR sample</th><th>Pigmentation</th><th>Fixation</th><th>Soaking after fixation</th><th>Diagnosis</th><th>Sex</th><th>Age</th></tr>
<tr><td>1</td><td>Pigmented</td><td>Formalin</td><td>No</td><td rowspan="4">AKU</td><td rowspan="4">Male</td><td rowspan="4">55 years</td></tr>
<tr><td>2</td><td>Pigmented</td><td>Formalin</td><td>Yes</td></tr>
<tr><td>3</td><td>Non-pigmented</td><td>Formalin</td><td>No</td></tr>
<tr><td>4</td><td>Non-pigmented</td><td>Formalin</td><td>Yes</td></tr>
<tr><td>5</td><td>Non-pigmented</td><td>Formalin</td><td rowspan="2">Yes</td><td rowspan="2">OA (proximal)
OA (distal)</td><td rowspan="2">Female</td><td rowspan="2">71 years</td></tr>
<tr><td>6</td><td>Non-pigmented</td><td>Formalin</td></tr>
</table>

Each NMR sample constitutes a separate 3.2 mm rotor. In all cases, knee articular cartilage was sampled.

For the OA samples, cartilage is taken from two parts of the femoral head from the same patient: *proximal* to the site where cartilage has been worn away, and *distal* from the cartilage damage site. As the DNP-enhanced NMR spectra from the two OA samples were very similar, only the sample taken distal to the cartilage damage site was shown in the figures.

NMR samples 1 and 2, prior to rotor packing, are shown in Supplementary Fig. S3.

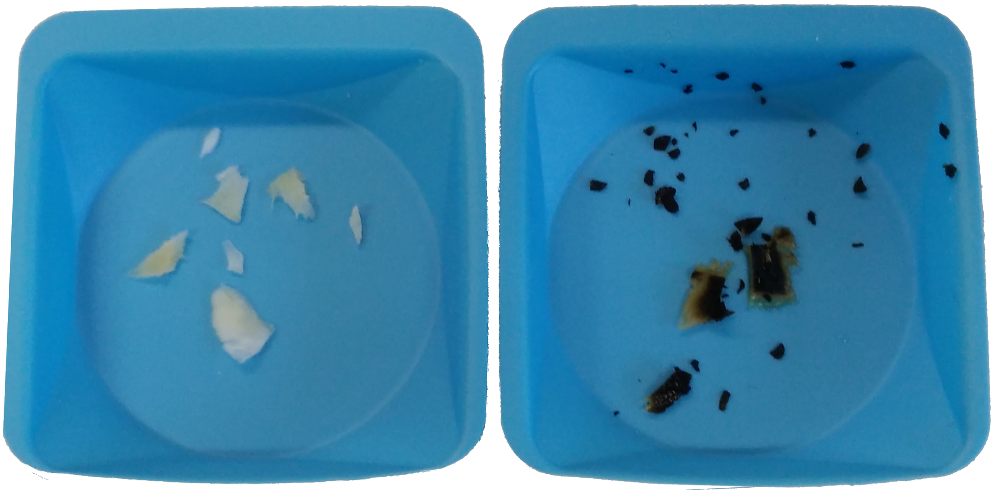

Supplementary Figure S3: Unpigmented and pigmented cartilage used for NMR spectroscopy in the current study. Both samples of cartilage were donated by the same AKU patient.

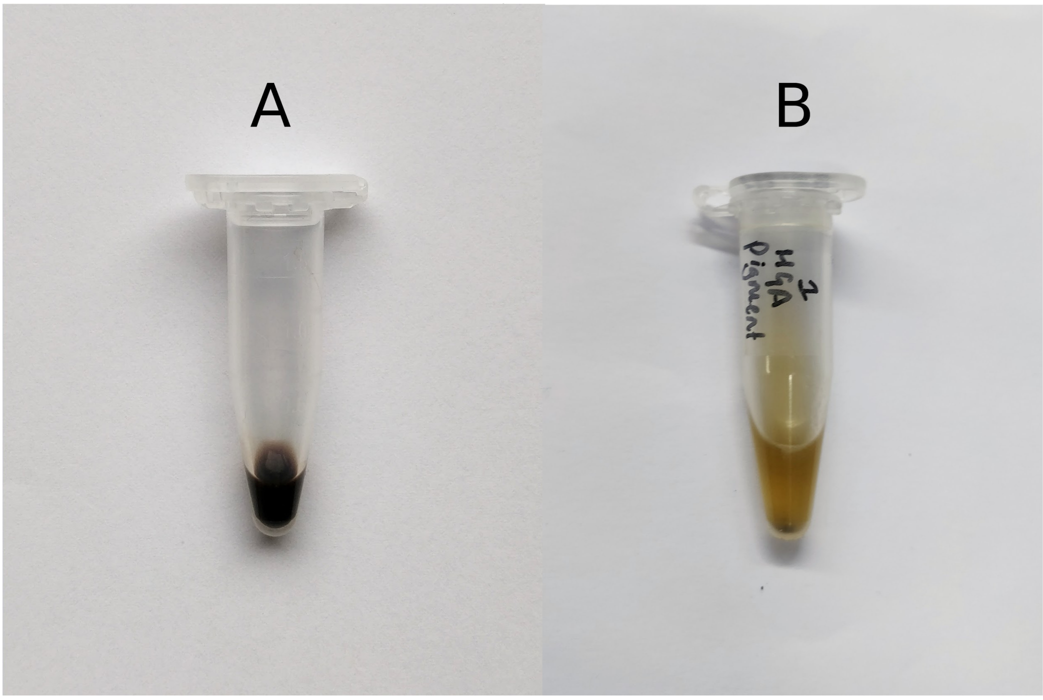

Supplementary Figure S4: Brown solution generated by alkaline oxidation of 10 mM HGA over 4 months under aerobic conditions. (A) dried, then dissolved again in a small amount of water, used for NMR and X-band EPR spectroscopy in the current study; (B) without drying, used for W-band EPR spectroscopy.

Supplementary Table S4: Mouse condyle sample details.

NMR sample	Genetic background	Fixation	Strain	Sex	Age
7	WT	None	BALB/c	Female	7 months
			BALB/c	Female	7 months
8	WT	Formalin	C57BL/6J	Female	15 months
			BALB/c	Female	7 months
9	$HGD^{-/-}$	None	C57BL/6J	Male	9 months
			C57BL/6J	Male	9 months
10	$HGD^{-/-}$	Formalin	C57BL/6J	Male	9 months
			BALB/c	Male	16 months

For the mouse samples, condyles collected from two individuals were pooled into one ssNMR rotor to increase the signal to noise ratio due to the very small sample amounts per animal.

The tissue obtained from the wild type (WT) and $HGD^{-/-}$ mice are similar in appearance macroscopically, and do not demonstrate the obvious pigmentation observed for the human AKU cartilage samples.

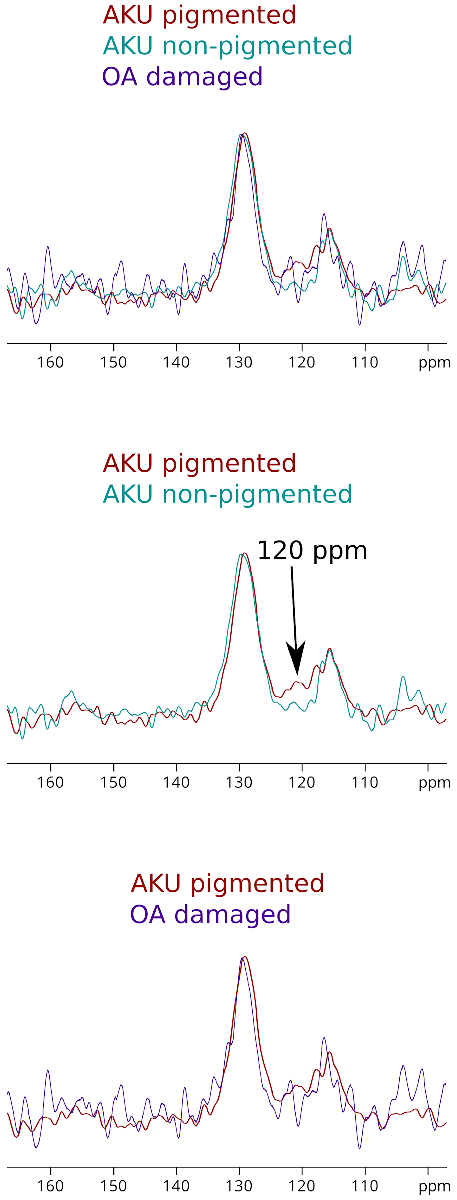

Supplementary Figure S5: ^{13}C spectra extracted from the 2D ssNMR ^{1}H-^{13}C FSLG HETCOR DNP-enhanced ssNMR spectra of pigmented AKU human cartilage (red), non-pigmented AKU human cartilage (green) and OA human cartilage (purple) at 50 μs contact times. To aid visual comparison, the top shows all three overlaid, the middle and bottom show just two out of the three same spectra overlaid. The vertical height of the largest signal in each spectrum were scaled to match each other. The AKU pigmented cartilage show a broadening/smearing of signal intensity at 120 ppm that is clearly absent from the AKU non-pigmented cartilage, and is also weak or absent from the OA damaged cartilage though it is harder to gauge due to the poor signal to noise of the OA cartilage spectrum even with DNP enhancement.

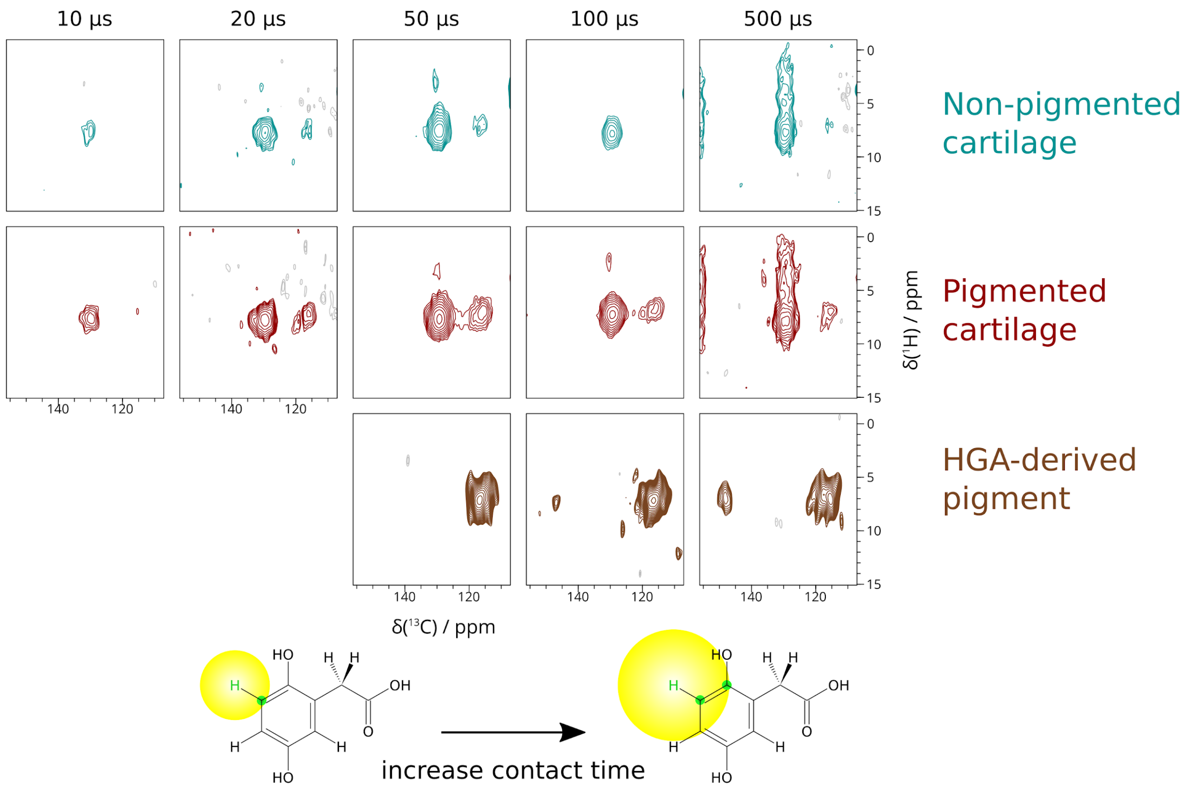

Supplementary Figure S6: ^{1}H-^{13}C FSLG HETCOR ssNMR spectra of **unsoaked** pigmented human cartilage (top), non-pigmented human cartilage (middle) and HGA-derived pigment (bottom) under DNP conditions at various contact times from 10 µs - 500 µs. Only the ^{13}C 105-155 ppm range is shown.

In the HGA-derived pigment experimental series (green) in Supplementary Fig. S6, while the contact time is increased from 50 µs to 500 µs, more signals were observed. The signal at ^{13}C 116.8 ppm correspond to carbons directly bonded to a proton in the aromatic ring, and is present in all spectra. In the 100 and 500 µs contact time spectrum (right 2 rows), an additional signal emerges at ^{13}C 148.5 ppm, corresponding to carbons that are directly bonded to a hydroxyl group (i.e. a phenyl carbon), where the rate of signal buildup is distinctly slower than the 116.8 ppm signal as the distance to the nearest proton is greater.

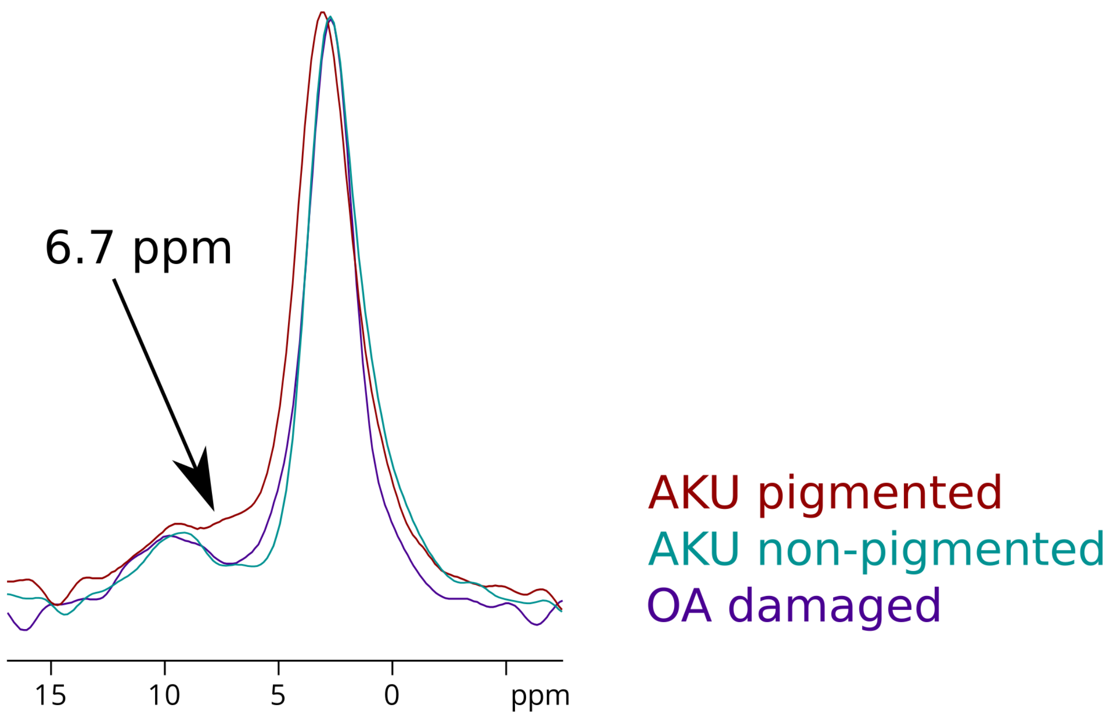

Supplementary Figure S7: ^{1}H spectra extracted from the 2D ssNMR ^{1}H-^{13}C FSLG HETCOR DNP-enhanced ssNMR spectra of pigmented AKU human cartilage (red, top), non-pigmented AKU human cartilage (green, middle) and OA human cartilage (purple, bottom) at 100 µs contact times. The vertical height of the largest signal in each spectrum were scaled to match each other. The AKU pigmented cartilage show a broadening/smearing of signal intensity at 6.7 ppm that is absent in the AKU non-pigmented and OA damaged cartilage.

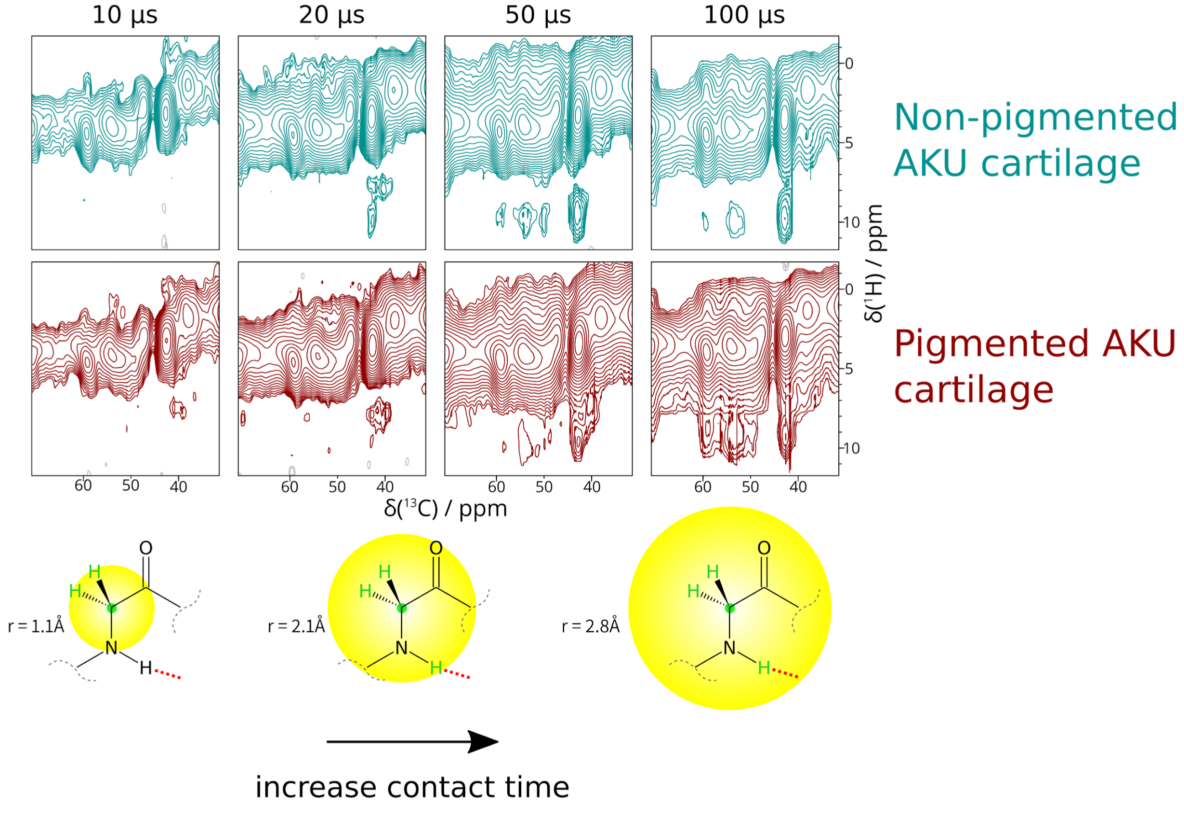

Supplementary Figure S8: ^{1}H-^{13}C FSLG HETCOR DNP-enhanced ssNMR spectra of **unsoaked** non-pigmented human cartilage (top, green) and pigmented human cartilage (bottom, red) at various contact times from 10 µs - 500 µs, plotting only the ^{13}C 30-70 ppm range.

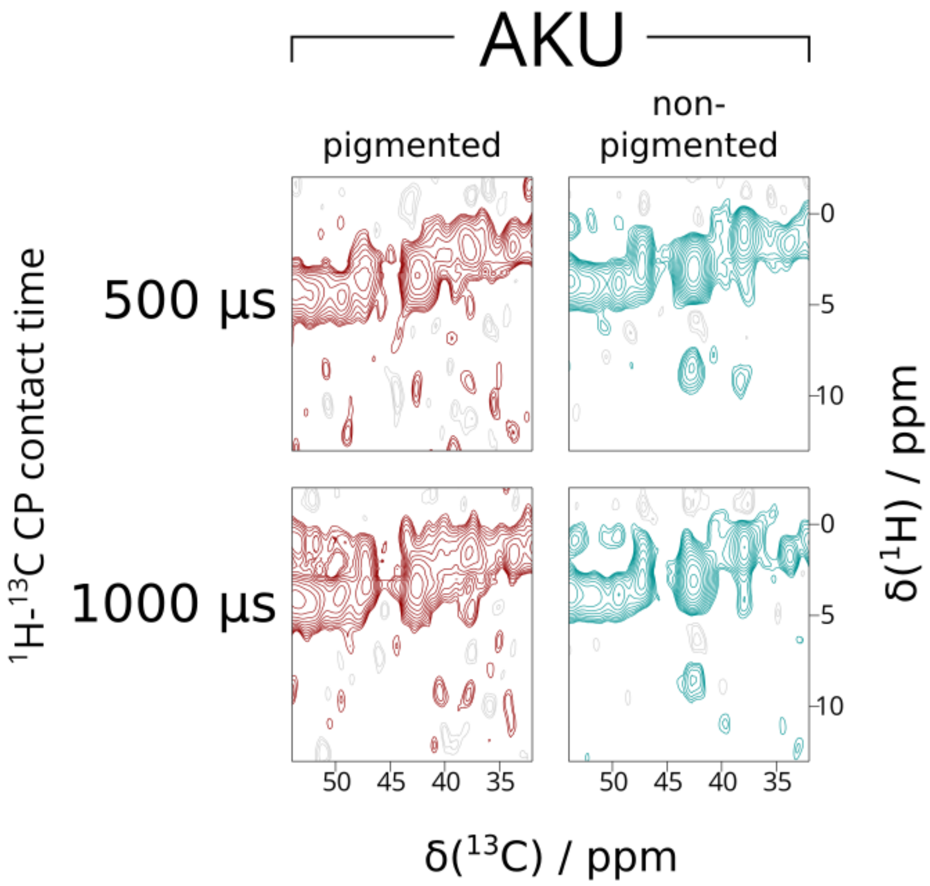

Supplementary Figure S9: ^{1}H-^{13}C FSLG HETCOR ssNMR spectrum of pigmented human cartilage (red, left) and non-pigmented human cartilage (green, right) at a contact time of 500-1000 µs, showing only the ^{13}C 30-55 ppm range. Both samples were soaked in water prior to the experiment to remove methylene glycol signals, but no radical solution was added. Sample temperature was 285 K.

References

[45] C. Sauvée, M. Rosay, G. Casano, F. Aussenac, R. T. Weber, O. Ouari, P. Tordo, *Angew. Chem. Int. Ed Engl.* **2013**, *125*, 11058–11061.
[46] A. P. Jagtap, M.-A. Geiger, D. Stöppler, M. Orwick-Rydmark, H. Oschkinat, S. T. Sigurdsson, *Chem. Commun.* **2016**, *52*, 7020–7023.
[47] M.-A. Geiger, A. P. Jagtap, M. Kaushik, H. Sun, D. Stöppler, S. T. Sigurdsson, B. Corzilius, H. Oschkinat, *Chemistry* **2018**, DOI 10.1002/chem.201801251.
[48] G. C. Na, *Biochemistry* **1986**, *25*, 967–973.
[49] A. Lesage, M. Lelli, D. Gajan, M. A. Caporini, V. Vitzthum, P. Miéville, J. Alauzun, A. Roussey, C. Thieuleux, A. Mehdi, et al., *J. Am. Chem. Soc.* **2010**, *132*, 15459–15461.
[50] A. Weidinger, M. Waiblinger, B. Pietzak, T. Almeida Murphy, *Appl. Phys. A: Mater. Sci. Process.* **1998**, *66*, 287–292.
[51] G. Metz, X. L. Wu, S. O. Smith, *J. Magn. Reson. A* **1994**, *110*, 219–227.
[52] G. A. Morris, R. Freeman, *J. Am. Chem. Soc.* **1979**, *101*, 760–762.
[53] B.-J. van Rossum, H. Förster, H. J. M. de Groot, *J. Magn. Reson.* **1997**, *124*, 516–519.
[54] J. P. Martin Jr, B. Batkoff, *Free Radic. Biol. Med.* **1987**, *3*, 241–250.
[55] R. A. Milch, E. D. Titus, T. L. Loo, *Science* **1957**, *126*, 209–210.
[56] R. Consden, H. A. W. Forbes, L. E. Glynn, W. M. Stanier, *Biochem. J* **1951**, *50*, 274–278.
[57] S. Yamaguchi, N. Koda, T. Ohashi, *Tohoku J. Exp. Med.* **1986**, *150*, 227–228.
[58] A. M. Taylor, K. P. Vercruysse, *JIMD Rep.* **2017**, *35*, 79–85.
[59] J. T. Mason, T. J. O'Leary, *J. Histochem. Cytochem.* **1991**, *39*, 225–229.
[60] A. J. Preston, C. M. Keenan, H. Sutherland, P. J. Wilson, B. Wlodarski, A. M. Taylor, D. P. Williams, L. R. Ranganath, J. A. Gallagher, J. C. Jarvis, *Ann. Rheum. Dis.* **2013**, *73*, 284–289.
[61] R. Thavarajah, V. K. Mudimbaimannar, J. Elizabeth, U. K. Rao, K. Ranganathan, *J. Oral Maxillofac. Pathol.* **2012**, *16*, 400–405.
[62] Q. Cui, I. A. Lewis, A. D. Hegeman, M. E. Anderson, J. Li, C. F. Schulte, W. M. Westler, H. R. Eghbalnia, M. R. Sussman, J. L. Markley, *Nat. Biotechnol.* **2008**, *26*, 162–164.
[63] N. Hayashi, T. Yoshikawa, T. Ohnuma, H. Higuchi, K. Sako, H. Uekusa, *Org. Lett.* **2007**, *9*, 5417–5420.
[64] T. M. Madkour, *Polym. J.* **1997**, *29*, 670–677.
[65] M. Fišer-Herman, M. Petrovački, *Clin. Chim. Acta* **1958**, *3*, 248–252.

Author Contributions

W.Y.C., B.P.N., J.A.G. conceived and designed the study. B.P.N., J.A.G., L.R.R. obtained ethical approval for usage and transfer of the patient cartilage tissue samples. B.P.N., J.A.G., L.R.R, N.B.R. proposed the successful iNext project to access NMR infrastructure (ID 1952) with support from W.Y.C. and H.O.. B.P.N. and W.Y.C. acquired the NMR data. C.T. acquired the EPR data. W.Y.C. processed NMR data with input from H.O.. All authors contributed to the analysis and interpretation of the data, with particular input from B.P.N., W.Y.C., N.B.R.. W.Y.C. and B.P.N. drafted the manuscript. All authors were involved with revisions of the manuscript, with particularly substantial revisions from M.J.D., H.O. and J.A.G.. W.Y.C. and B.P.N. contributed equally to the work.
